# SEPARATED KIN: RURAL-URBAN MIGRATION AND MENTAL HEALTH TRAJECTORIES OF OLDER ADULTS LEFT BEHIND IN RURAL CHINA

**DOI:** 10.1093/geroni/igz038.3223

**Published:** 2019-11-08

**Authors:** Zhiyong Lin

**Affiliations:** University of Maryland, College Park, Maryland, United States

## Abstract

As the processes of urbanization and globalization have intensified across the world, a burgeoning literature has documented the impact of emigration on the health of family members left behind in emigrant communities. Although the association between children’s migration and parental well-being is well documented, few have examined the health implications of children’s migration in the milieu of multiple children and further differentiated between children’s short-term and long-term migration. Therefore, I argue that it is not the geographic locality of a single child but the composition of all children’s location that matters. I further suggest that the impact of children’s migration on parental wellbeing is conditioned on the duration of children’s migration. Using a six waves longitudinal data (2001-2015) collected in rural China, this paper compares mental health (measured as depressive symptoms) trajectories of old adults (aged 60 and older) across different compositions of local and migrant children over a 14-year span. Results from growth curve models show that parents having more migrant children relative to local children experience a more rapid increase in depressive symptoms. In addition, older adults who have their most children migrate away for three or more waves of data have experienced the steepest rate of increase in depressive symptoms. These findings provide new evidence to support the life course processes of mental health disparities among older adults from the perspective of intergenerational proximity.

